# Macrophage migration inhibitory factor receptor CD74 expression is associated with expansion and differentiation of effector T cells in COVID-19 patients

**DOI:** 10.3389/fimmu.2023.1236374

**Published:** 2023-10-25

**Authors:** Jaana Westmeier, Annika Brochtrup, Krystallenia Paniskaki, Zehra Karakoese, Tanja Werner, Kathrin Sutter, Sebastian Dolff, Andreas Limmer, Daniela Mittermüller, Jia Liu, Xin Zheng, Tetiana Koval, Igor Kaidashev, Marc Moritz Berger, Frank Herbstreit, Thorsten Brenner, Oliver Witzke, Mirko Trilling, Mengji Lu, Dongliang Yang, Nina Babel, Timm Westhoff, Ulf Dittmer, Gennadiy Zelinskyy

**Affiliations:** ^1^ Institute for Virology, University Hospital Essen, University of Duisburg-Essen, Essen, Germany; ^2^ Department of Infectious Diseases, West German Centre of Infectious Diseases, University Hospital Essen, University Duisburg-Essen, Essen, Germany; ^3^ Center for Translational Medicine, Medical Department I, Marien Hospital Herne, University Hospital of the Ruhr-University Bochum, Herne, Germany; ^4^ Joint International Laboratory of Infection and Immunity, Huazhong University of Science and Technology (HUST), Wuhan, China; ^5^ Department of Anesthesiology, University Hospital Essen, University Duisburg-Essen, Essen, Germany; ^6^ Department of Pediatric Heart Surgery, Friedrich-Alexander- Universität Erlangen-Nürnberg, Erlangen, Germany; ^7^ Department of Infectious Diseases, Union Hospital of Tonji Medical College, Huazhong University of Science and Technology (HUST), Wuhan, China; ^8^ Department of Infectious Diseases with Epidemiology, Poltava State Medical University, Poltava, Ukraine; ^9^ Department of Internal Medicine №3 with Phthisiology, Poltava State Medical University, Poltava, Ukraine; ^10^ Charité – Universitätsmedizin Berlin, Corporate Member of Freie Universität Berlin, Humboldt-Universität zu Berlin, and Berlin Institute of Health, Berlin-Brandenburg Center for Regenerative Therapies, Berlin, Germany; ^11^ Medical Department I, Marien Hospital Herne, University Hospital of the Ruhr University of Bochum, Herne, Germany

**Keywords:** COVID-19, SARS-CoV-2, cytotoxic T cells, CD8+, CD4+, MIF, CD74

## Abstract

Severe acute respiratory syndrome coronavirus 2 (SARS-CoV-2) caused millions of COVID-19 cases and deaths worldwide. Severity of pulmonary pathologies and poor prognosis were reported to be associated with the activation non-virus-specific bystander T cells. In addition, high concentrations of the macrophage migration inhibitory factor (MIF) were found in serum of COVID-19 patients. We hypothesized that these two pathogenic factors might be related and analyzed the expression of receptors for MIF on T cells in COVID-19. T cells from PBMCs of hospitalized patients with mild and severe COVID-19 were characterized. A significantly higher proportion of CD4+ and CD8+ T cells from COVID-19 patients expressed CD74 on the cell surface compared to healthy controls. To induce intracellular signaling upon MIF binding, CD74 forms complexes with CD44, CXCR2, or CXCR4. The vast majority of CD74+ T cells expressed CD44, whereas expression of CXCR2 and CXCR4 was low in controls but increased upon SARS-CoV-2 infection. Hence, T cells in COVID-19 patients express receptors that render them responsive to MIF. A detailed analysis of CD74+ T cell populations revealed that most of them had a central memory phenotype early in infection, while cells with an effector and effector memory phenotype arose later during infection. Furthermore, CD74+ T cells produced more cytotoxic molecules and proliferation markers. Our data provide new insights into the MIF receptor and co-receptor repertoire of bystander T cells in COVID-19 and uncovers a novel and potentially druggable aspect of the immunological footprint of SARS-CoV-2.

## Highlights

The antigen-independent activation of bystander T cells in viral infections gives rise to a population of cytotoxic, but non-specific T cells. Their action might contribute to virus control, but can also lead to immunopathologies. Viral infections and cellular stress result in the secretion of MIF. COVID-19 patients showed high MIF plasma levels and the expression of MIF receptor molecules on T cells. Depending on the stage of the disease, SARS-CoV-2 infection induced the expression of MIF receptors on different subpopulations of T cells. MIF receptor-expressing T cells were highly cytotoxic and proliferative. Thus, augmented MIF signaling might be a regulatory mechanism for the activation of bystander T cells in COVID-19 and may contribute to disease progression and severity.

## Introduction

Infections with the Severe Acute Respiratory Syndrome Coronavirus 2 (SARS-CoV-2), the infectious agent causing COVID-19, led to a global pandemic with more than 750 million cases and more than 7 million fatalities. Virus replication in the lung epithelium and a subsequent pneumonia are often associated with symptomatic COVID-19 cases, although other tissues and organs such as the brain, kidneys or the neuronal system can also be affected (1). The infection of lung epithelial cells and the resulting immune response against the virus lead to acute progressive pneumonia, which is a reason for reduced oxygen saturation in the blood and promotes the rapid disease progression in COVID-19 patients.

The death of infected lung epithelial cells triggers the production of macrophage inflammatory protein 1α (MIP1α), MIP1β, monocyte chemoattractant protein 1 (MCP1), interleukin 6 (IL-6), and Interferon gamma-induced protein 10 (IP-10) in neighboring cells (2). These proteins promote inflammation and migration of myeloid and lymphoid cells into infected compartments and, these cells also produce multiple cytokines. IL-2, IL-7, IL-10, granulocyte colony-stimulating factor (G-CSF), IP-10, MCP1, macrophage inflammatory protein 1α (MIP1α), and tumor necrosis factor (TNF) are elevated in the serum in severe cases of COVID-19 (3). A longitudinal analysis of SARS-CoV-2-infected patients shows a positive correlation between disease progression and the concentration of Macrophage Migration Inhibitory Factor (MIF) in the serum of patients (4). Increased concentrations of MIF as a pathogenetic inflammatory factor were also observed in patients with asthma and lung fibrosis (5).

MIF is a homotrimeric molecule with multiple functions, including an enzymatic activity, counter-regulation of immunosuppressive glucocorticoids, or pro-inflammatory action by recruitment of haemopoietic cells to sites of tissue damage (6, 7). MIF also sustains the pulmonary inflammation in the acute respiratory distress syndrome (ARDS) (8). In different inflammatory diseases of lung, levels of circulating MIF are elevated (9), raising the intriguing question if this molecule is involved in the pathogenesis of COVID-19 and/or Long-COVID. A polymorphism analysis of MIF promoter alleles revealed an association between COVID-19 progression and the enhanced expression of a distinct allele of this molecule in patients (10). Binding of MIF to CD74, which is expressed on the surface of immune cells, is necessary for signal transduction (11). CD74 is a single-pass type II membrane protein, which is also known as the MHC class II chaperone invariant chain (9). CD74 lacks an intrinsic intracellular signaling domain, and dependents on complex-formation with its co-receptors CD44, CXCR2, and CXCR4 to induce intracellular signaling (12–14). The cell-specific surface expression of the MIF receptor CD74 and its co-receptors defines the cellular responsiveness to MIF. The CD74/co-receptor signaling ultimately leads to the activation of the transcription factor NF-κB (15). While CD74 is mainly expressed on MHCII+ antigen-presenting cells, it can also be found on other cells derived from hematopoietic lineages such as T cells (16–18). Nevertheless, the role of MIF in SARS-CoV-2 infections remains elusive.

Virus-specific CD4+ and CD8+ T cells recognize different structural- and non-structural epitopes of SARS-CoV-2 (19). Both CD4+ and CD8+ T cells are necessary for viral control. Accordingly, cellular or functional T-cell deficiencies are associated with severe COVID-19 disease courses (20). However, an uncontrolled activity of these cells can lead to inflammation in infected organs and subsequent severe pathologies. A longitudinal transcriptome analysis observed a correlation between COVID-19 severity, the magnitude of bystander CD8+ T cell activation, and the production of pro-inflammatory and cytotoxic molecules by these cells (21). Bystander CD8+ T cells do not respond to viral antigens but to antigen-independent signals such as inflammatory cytokines (22). Thus, these two pathogenic factors in COVID-19, MIF and activated bystander CD8+ T cells may have simultaneously effects on disease progression and lethality. In our study, we characterized CD4+ and CD8+ T cells for their expression of receptors recognizing MIF in order to define the role of this soluble mediator in COVID-19 immunopathology and determine the interplay between these two pathogenic mechanisms. We observed that differentiated cytotoxic CD8+ T cells with central memory (CM), effector memory (EM) and effector phenotype showed enhanced expression of CD74 during SARS-Cov-2 infection. Simultaneously, COVID-19 patients presented high plasma levels of MIF. CD74-expressing cells showed enhanced proliferation and produced cytotoxic effector molecules such as granzymes. Thus, enhanced MIF concentrations were associated with the proliferation as well as functionality of bystander CD8+ T cells, and MIF might be an important biomarker of immunopathology in severe COVID-19.

## Materials and methods

### Study population and design

In this study, we recruited 81 patients with mild (n=39, 18 female, 21 male, median age 64) and severe (n=42, 13 female, 29 male, median age 54) COVID-19 immediately after admission to the hospital (**Supplement 1**). On average, hospitalization occurred one week after symptom onset. All COVID-19 patients included in this study had at least one positive SARS-CoV-2 PCR result (SARS-CoV-2 test, Altona Diagnostics, Hamburg). The severity of COVID-19 was determined according to WHO recommendations. The study was approved by the ethical committee of the Medical Faculty of the University Hospital Essen (ethics vote 20-9216-BO) and written informed consent was obtained from all study participants. Clinical characteristics of patients are shown in **Supplement 1**. As healthy controls, 19 age-matched uninfected individuals (15 female, 4 male) with a median age of 57 years were recruited.

### Isolation of PBMCs

Within the first 24 hours of hospitalization and on the seventh day of hospitalization, blood was drawn from COVID-19 patients with either a mild or severe disease course and collected in EDTA S-monovettes. Disease severity was grouped on the basis of the requirement for supplementary oxygen or ventilation (mild, hospitalized, no supplementary oxygen; severe, hospitalized, supplementary oxygen). Following separation of plasma and blood cells, peripheral blood mononuclear cells (PBMCs) were isolated from the latter as previously described (23). For cryopreservation, PBMCs were resuspended in FCS + 10% (v/v) DMSO, transferred to -80°C and stored in liquid nitrogen until further analyses.

### Quantification of MIF and sCD74 plasma levels

In order to determine concentrations of circulating MIF and soluble CD74, patient plasma was used to perform enzyme-linked immunosorbent assays (ELISAs). Plasma samples were diluted according to the manufacturer’s protocol. Levels of circulating MIF were quantified using a commercially available assay kit (R&D Systems) with a lower detection limit at 31.25 pg/ml. Plasma levels of sCD74 were also assayed by a commercial ELISA kit (Novus Biologicals) with a sensitivity of 0.38 ng/ml.

### *Ex vivo* stimulation of T cells with peptides and following staining for the detection of SARS-CoV-2-specific T cells by flow cytometry

PBMCs were incubated after thawing overnight in RPMI-1640 supplemented with 10% heat-inactivated fetal calf serum, 2 mM L-glutamine, 100 U/ml penicillin, 100 μg/ml streptomycin, at 37°C. The next day 8× 105PBMCs per well were plated in 96-wells U bottom. Cells were then stimulated with SARS-CoV-2 PepTivator S2, N, or M overlapping peptide pools (Miltenyi Biotec) overnight at 37°C. Positive controls were stimulated with PepTivator^®^ CEF MHC Class I Plus. For the last four hours Brefeldin was added to inhibit the secretion of cytokines.

Stimulated cells were washed with PBS and stained extracellularly for 20 min at room temperature in the staining buffer (PBS supplemented with 2% FCS) with fluorescently conjugated antibodies recognizing human CD3 (UCHT1, Biolegend), CD4 (OKT4, BD Biosciences), CD8 (53-6.7, BD Biosciences) and with Zombie Violet (Biolegend). Subsequently, the cells were washed with staining buffer and stained intracellular accordingly to protocol for eBioscience™ Foxp3/Transcription Factor Staining Set using fluorescently labeled antibodies to human: IL2 (MQ1-17H12; Biolegend), IFNγ (B27; Biolegend), TNFα (Mab11, BD Biosciences) for 45 min at room temperature. Finally, cells were washed with PBS and acquired on FACSymphony (BD Bioscience).

### Cell staining for flow cytometry analyses

Cell surface and intracellular antibody staining were performed as previously described (23). The cell surface levels were stained using antibodies recgnizing human CD3 (BW264/56, Miltenyi Biotec), CD4 (OKT4, BD), CD8 (2ST8.5H7, BD), CCR7 (G043H7, BioLegend), CD45RO (UCHL1, BioLegend), CD28 (CD28.2, BioLegend), CD25 (M-A251, BioLegend), CD127 (A019D5, BioLegend), CD44 (IM7, eBioscience), CXCR2 (5E8/CXCR2, BioLegend), CXCR4 (12G5, BioLegend), and CD74 (5-329, Miltenyi Biotec). For intracellular staining, antibodies specific for human GzmA (CB9, BioLegend), GzmB (QA16A02, BioLegend), GzmK (GM26E7, BioLegend), and perforin (B-D48, BioLegend) were used. To exclude dead cells from the analysis, Zombie UV Fixable Viability dye (BioLegend) was used.

Data acquisition was performed on a FACSymphony A5 Cell Analyzer (BD) with 25,000 to 100,000 lymphocyte-gated events per measured sample. Acquired data were analyzed using FACSDiva (BD) and FlowJo softwares (BD). Absolut numbers of CD3+CD4+CD74+ and CD3+CD8+CD74 T cells in the blood were calculated from lymphocytes counts determined in a certified clinical laboratory for every patient.

### Statistical analysis

When comparing three groups, the Kruskal-Wallis test with Dunn’s multiple comparison test was applied. A comparison of two groups was performed using the Mann-Whitney-U-test. Statistical analyses comparing samples from two time points of infection were performed using a one-tailed paired t-test (GraphPad Prism software; GraphPad Software, Inc.).

## Results

### MIF and sCD74 plasma concentrations are enhanced in COVID-19 patients

**Figure 1:**
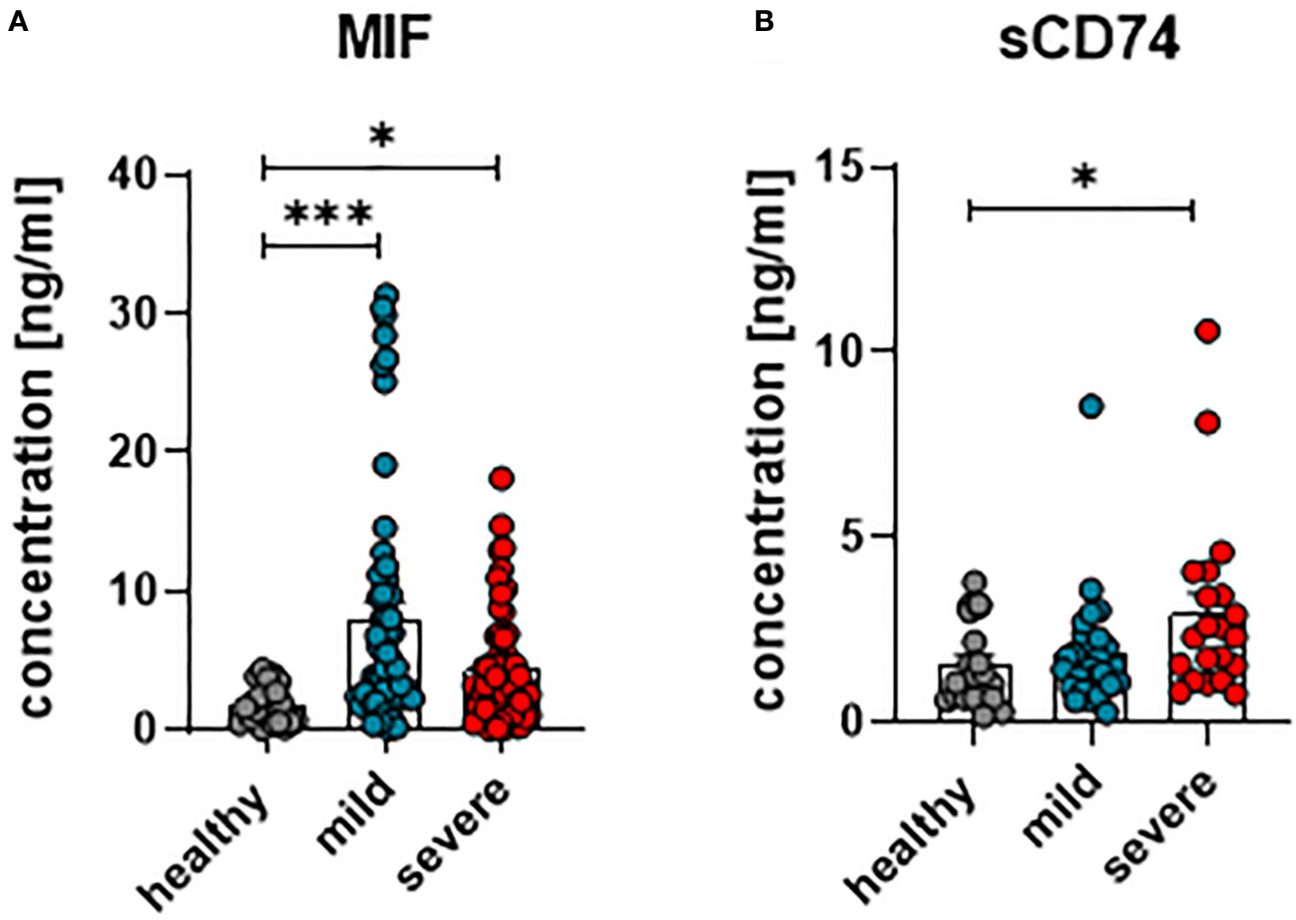
Concentrations of plasma MIF and sCD74 in COVID-19. The concentration of MIF **(A)** and sCD74 **(B)** in the plasma of patients with mild or severe COVID-19 and healthy donors were measured by ELISA. Each dot represents an individual patient. Statistically significant differences are indicated by asterisks (* < 0.05; *** < 0.001).

In order to investigate whether MIF and its receptor CD74 are involved in the immune regulation during anti-SARS-CoV-2 immune responses, concentrations of MIF and soluble CD74 (sCD74) were quantified in the plasma of patients with mild or severe courses of COVID-19, and compared with healthy controls. Within the first 24 hours of hospitalization, blood was collected from COVID-19 patients with different disease severities. To determine concentrations of MIF and sCD74, plasma levels were determined by ELISA. Overall, levels of MIF were significantly increased in COVID-19 patients in comparison to age- and sex-matched healthy controls, while disease severity did not have an effect on MIF concentrations (mean MIF concentrations: healthy 2.41 ng/ml; mild 8.04 ng/ml and severe 4.44 ng/ml) (**Figure 1A**). Although both COVID-19 disease courses resulted in increased plasma concentrations of sCD74, the soluble form of the MIF receptor, only severe COVID-19 resulted in significantly elevated sCD74 levels compared to healthy controls (mean sCD74 concentrations: healthy 0.6 ng/ml; mild 1.55 ng/ml and severe 2.7 ng/ml) (**Figure 1B**). Thus, our data confirm the enhancement of MIF upon SARS-CoV-2 infection and also show elevated sCD74 plasma levels in COVID-19 patients. The data proposes a possible regulatory effect of MIF on T cell responses during COVID-19.

### SARS-CoV-2 infection alters MIF receptor and co-receptor expression on T cells

**Figure 2:**
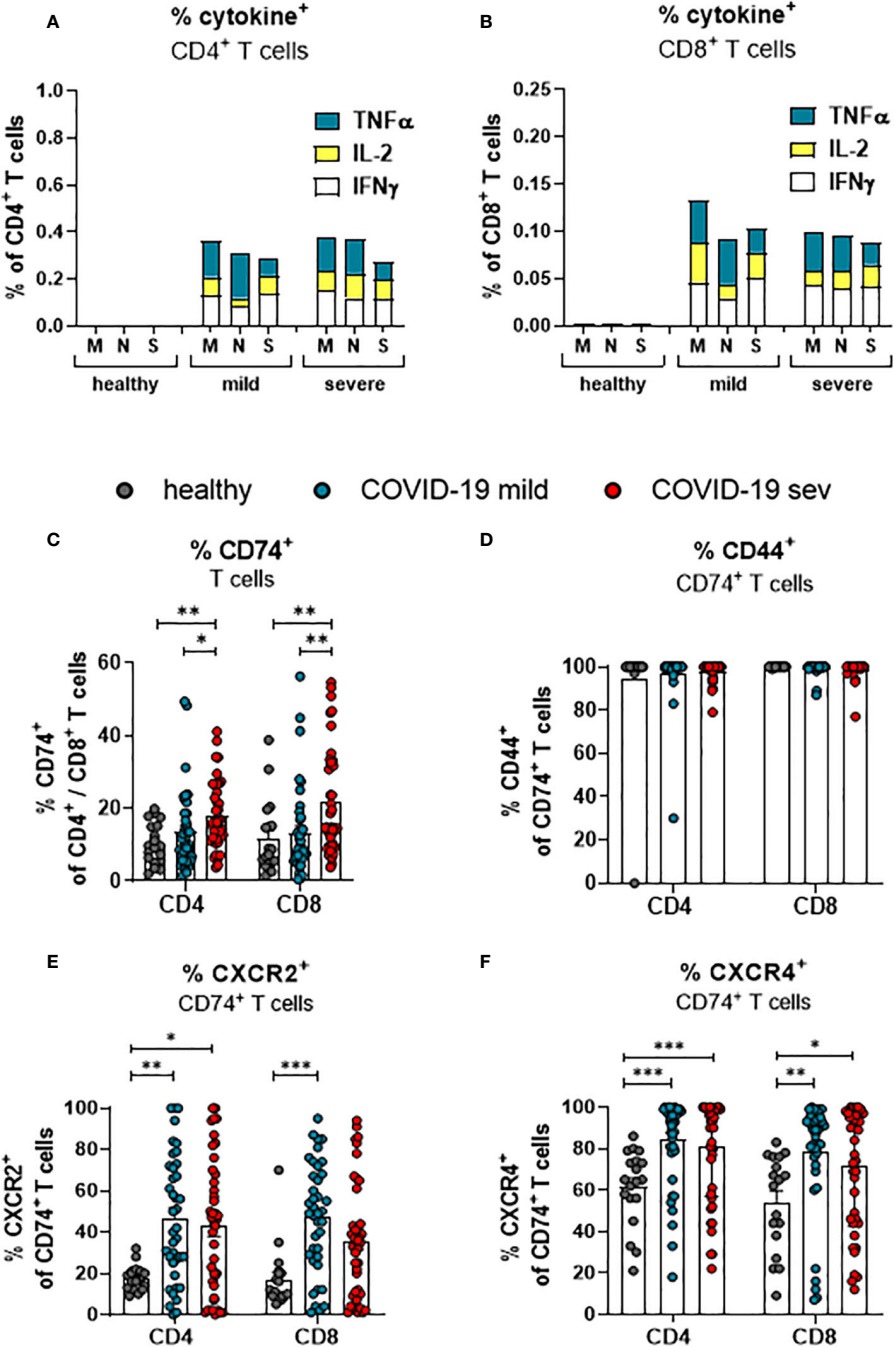
SARS-CoV-2-specific and MIF receptor- and co-receptor positive T cells. Frequencies of TNFa (blue), IL-2 (yellow), and IFNy (white) expressing CD4^+^ **(A)** and CD8^+^ **(B)** T cells from mildly or severely ill COVID-19 patients and healthy controls after stimulation with SARS-COV-2 M, N or S1 protein peptides were determined by flow cytometric analysis. Percentages of total CD4^+^ and CD8^+^ T cells from mildly (mild) or severely (sev) diseased patients and healthy donors expressing CD74 **(C)**, CD74 and CD44 **(D)**, CD74 and CXCR2 **(E)** or CD74 and CXCR4 **(F)** were analyzed by flow cytometry. Each dot represents an individual patient. Statistically significant differences are indicated by asterisks (* < 0.05; ** < 0.01; *** < 0.001).

Virus-specific CD4+ and CD8+ T cells recognize antigen-expressing cells and apply multiple mechanisms for the suppression of virus replication and elimination of infected cells. In order to determine frequencies of SARS-CoV-2-specific T cells in COVID-19 patients, PBMCs were stimulated with peptide pools derived from the SARS-CoV-2-endoded proteins spike (S), membrane protein (M), and nucleocapsid protein (N) before the percentages of cells producing TNFα, IL-2, and IFNγ was determined. The frequencies of CD4+ (**Figure 2A**) and CD8+ (**Figure 2B**) T cells that showed a specific response to the individual peptide pools were rather low. For both CD4+ and CD8+ T cells, the frequency of all circulating SARS-Cov-2 specific T cells in the blood comprised less than one percent of the corresponding total population. A similar low frequency of virus-specific T cells was observed in other studies (19, 24). However, the frequency of CD8+ and CD4+ T cells with effector or memory phenotype in COVID-19 is much higher than the frequency of SARS-CoV-2 specific T cells (21, 25). These cells most likely encountered other antigens before and are then re-activated during COVID-19 in an antigen-independent manner, possibly by inflammatory cytokines. The current study focuses on the characterization of total T cell populations, most of which cells are bystander T cells.

To this end, we determined the frequencies of total T cells that expressed receptors or co-receptors for MIF by multiparameter flow cytometry. The frequency of CD74-expressing cells was slightly higher in the population of CD8+ T cells than in the population of CD4+ T cells isolated from healthy individuals (11.6% and 10.4%, respectively; **Figure 2C**). Upon SARS-CoV-2 infection, frequencies of CD74-expressing CD4+ T cells from COVID-19 patients with a mild disease course were not altered in comparison to healthy individuals. Increased proportions of CD74+ CD4+ T cells were only detected in severely ill patients (mean: healthy 10.4%, mild 13.5%, severe 17.8%). A mild disease course did also not lead to a significant increase in the frequencies of CD74+ CD8+ T cells, whereas severe COVID-19 was associated with increased percentages of CD74+ CD8+ T cells (mean: healthy 11.6%, mild 12.4% severe 21.7%; **Figure 2C**). In addition to the frequencies, absolute numbers of CD74+CD4+ and CD74+CD8+ T cells per µl of blood were calculated (**Supplement 2A**). CD74+ T cell numbers were similar in all characterized groups. The most likely explanation for this difference to the frequencies of CD74+ cells was that most patients present with lymphopenia during the acute phase of SARS-CoV-2 infection (26). The MIF receptor CD74 lacks an intracellular signaling domain. For intracellular signaling, CD74 interacts with its co-receptors CD44, CXCR2, or CXCR4. Accordingly, staining for the co-expression of CD74 and its co-receptors on T cells shows whether an infection with SARS-CoV-2 induce signaling in T cells. First, we determined the frequencies of CD44 co-expressing CD74+ CD4+ T cells and CD74+ CD8+ T cells. Even in healthy controls, nearly all CD74+ cells co-expressed CD44, and frequencies of CD74+ T cells did not change during SARS-CoV-2 infection (**Figure 2D**). This finding indicates that almost all CD74+ T cells can respond to MIF binding through the CD44 signaling domain. In addition, we found that the expression of other CD74 co-receptors was also altered during SARS-CoV-2 infection. For CD4+ T cells, an increase in frequencies of CXCR2+ CD74+ cells was found in both mild and severe COVID-19 compared to healthy controls (mean: healthy: 17.7%, mild 46.9%, severe 42.9%). For CD8+ T cells, significant differences were restricted to a mild disease course in comparison to healthy controls (mean: healthy 17.0%, mild 47.3%, severe 35.7%; **Figure 2E**). Also, the co-expression of CD74 and CXCR4 on T cells was analyzed. About 61.5% of the CD74+ CD4+ T cells and 54.2% of the CD74+ CD8+ T cells in healthy donors expressed CXCR4, and SARS-CoV-2 infection enhanced the CXCR4 expression on T cells. Significantly increased percentages of CXCR4-expressing CD74+ CD4+ T cells (mean: healthy 61.5%, mild 84.0%, severe 81.0%; **Figure 2F**) and CXCR4 CD74+ CD8+ T cells (mean: healthy 54.2%, mild 78.4%, severe 71.7%; **Figure 2F**) were observed in both groups of COVID-19 patients in comparison to healthy controls. Again, these differences were not found for absolute numbers of CD74+ T cells that simultaneously expressed coreceptor molecules because of the severe lymphopenia in COVID-19 patients (**Supplement 2B, C**).

Taken together, COVID-19 is associated with an enhanced expression of the MIF receptor CD74 on total CD4+ and CD8+ T cells independent of the disease severity. While CD74+ T cells constitutively expressed CD44, SARS-CoV-2 enhances the expression of the co-receptors CXCR2 and CXCR4 on T cells.

### Frequencies of CD74^+^ convCD4^+^ T cells, but not Tregs, are increased upon SARS-CoV-2 infection

**Figure 3:**
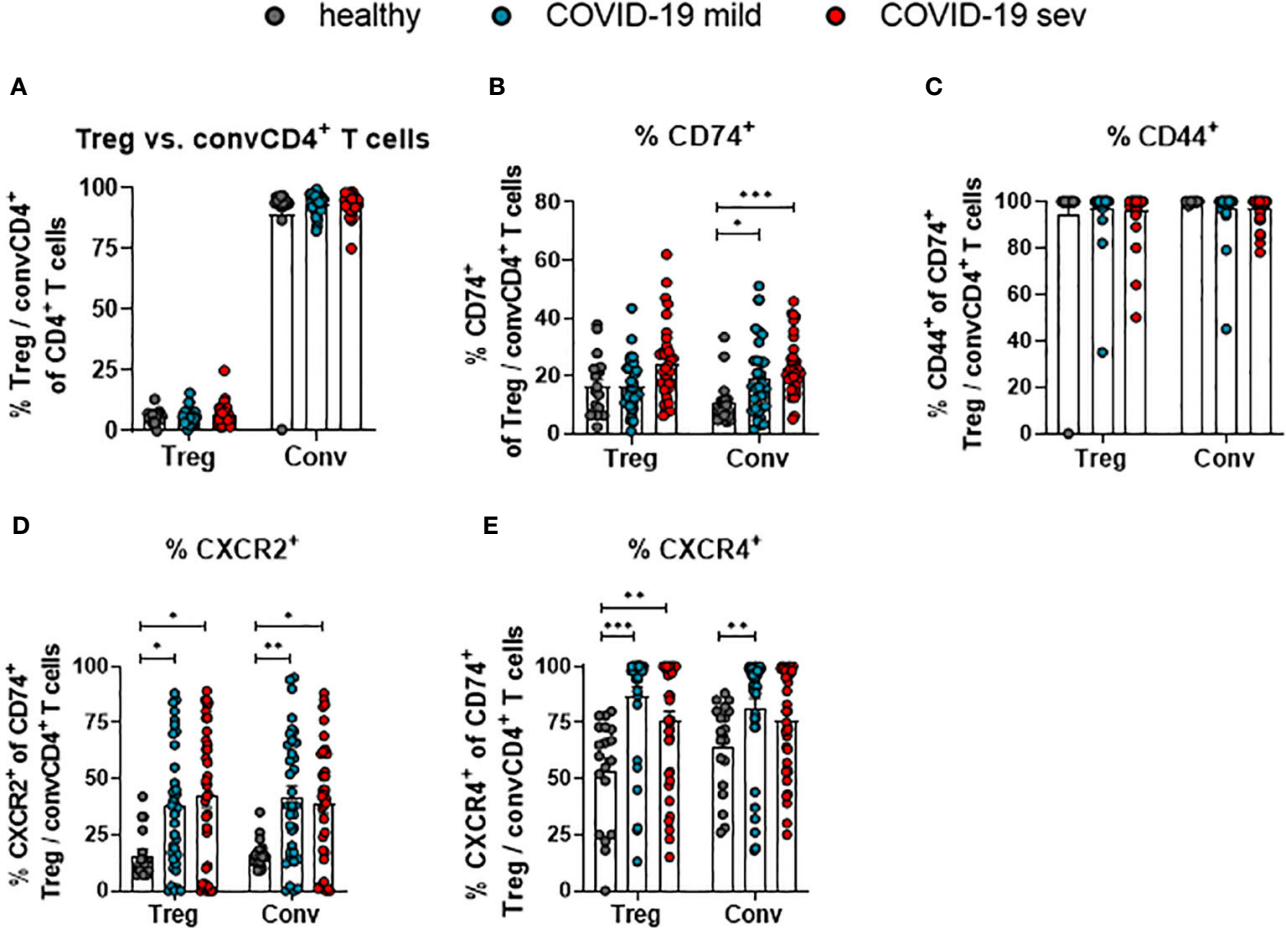
Receptor and co-receptor positive Treg and convCD4^+^ T cells. Percentages of Treg and convCD4^+^ (conv) T cell subpopulations **(A)** from mildly (mild) or severely (sev) ill COVID-19 patients and healthy donors; frequencies of CD74^+^ Treg or convCD4^+^ T cells **(B)**, CD44+ CD74+ Treg or convCD4^+^ T cells **(C)**, CXCR2^+^ CD74^+^ Treg or convCD4^+^ T cells **(D)** and CXCR4^+^ CD74^+^ Treg or convCD4^+^ T cells **(E)** were analyzed by flow cytometry. Treg (CD127^−^ CD25^+^ CD4^+^) and convCD4^+^ T cells (CD127^+^ CD25^−^ CD4^+^) were characterized by using CD127 and CD25. Each dot represents an individual patient. Statistically significant differences are indicated by asterisks (* < 0.05; ** < 0.01; *** < 0.001; Kruskal-Wallis test with Dunn's multiple comparisons test).

CD4+ T cells are a very heterogeneous population. Most prominent functional differences are observed between regulatory T cells (Treg; CD127- CD25+ CD4+) and conventional CD4+ T cells (convCD4+; CD127+ CD25+ CD4+) (18). In order to define which of these subpopulations of CD4+ T cells might be regulated by MIF, their receptor expression was compared. No changes in the overall proportions of Tregs and convCD4+ T cells were detectable upon SARS-CoV-2 infection (**Figure 3A**). The analysis of CD74 on CD4+ cells revealed an overall higher percentage of CD74+ Tregs compared to convCD4+ T cells in healthy controls (mean: 16.3% and 10.7% CD74+ cells, respectively; **Figure 3B**). Frequencies of CD74+ Tregs did not significantly change during SARS-CoV-2 infection, while percentages of CD74+ convCD4+ T cells increased upon SARS-CoV-2 infection (mean: healthy 10.7%, mild 18.9%, severe 22.9%; **Figure 3B**). During SARS-CoV-2 infection, frequencies of cells that co-express CD74 and CD44 were always very high and did not change on Tregs or convCD4+ T cells (**Figure 3C**). In contrast, the expression of CXCR2 on CD74+ Tregs (mean: healthy 15.9%, mild 37.5%, severe 42.4%) as well as on CD74+ convCD4+ T cells (mean: healthy 16.3%, mild 42.1%, severe 39.1%) was enhanced in COVID-19 patients (**Figure 3D**). ConvCD4+ T cells with co-expression of CXCR4 and CD74 showed a significant difference between healthy controls and mildly ill patients (mean: healthy 64.5%, mild 81.5%, severe 76.0%; **Figure 3E**), while frequencies of CXCR4+ CD74+ Tregs were increased irrespective of the disease severity (mean: healthy 53.5%, mild 86.8%, severe 75.6%; **Figure 3E**). Thus, convCD4+ T cells upregulated CD74 and its co-receptors upon SARS-CoV-2 infection, whereas Tregs predominantly upregulated the MIF-binding chemokine co-receptors.

### During COVID-19, levels of CD74 and its co-receptors are enhanced on convCD4+ T cells with a memory phenotype

**Figure 4:**
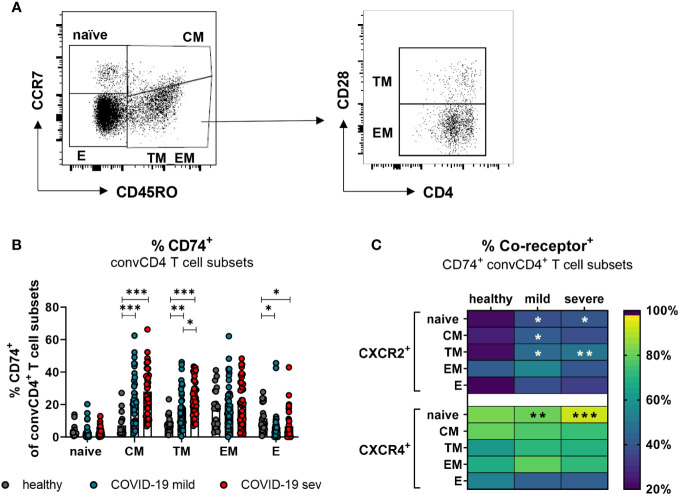
CD74 and co-receptor surface expression on convCD4^+^ T cell subpopulations. Naïve (CCR7^+^CD45RO^−^CD28^+^), central memory (CM, CCR7^+^CD45RO^+^CD28), transitional memory (TM, CCR7^−^CD45RO^+^CD28^+^), effector memory (EM, CCR7^−^CD45RO^+^CD28^−^), and effector (E, CCR7-CD45RO^−^CD28^−^) T cell subpopulations were characterized by using CD45RO, CCR7, and CD28 **(A)**. Frequencies of CD74^+^ convCD4^+^ T cell subsets **(B)** were measured. Each dot represents an individual patient. Percentages of co-receptor positive CD74^+^ convCD4^+^ T cells are depicted in **(C)** for CXCR2^+^ and CXCR4^+^ cells. Statistically significant differences are indicated by asterisks (* < 0.05; ** < 0.01; *** < 0.001).

T lymphocytes circulating in the blood belong to different stages of cell differentiation. In order to determine the association between the expression of CD74 and its co-receptors with the differentiation status of convCD4+ T cells from COVID-19 patients, flow cytometric analyses were performed. ConvCD4+ T cells were stratified into naïve (CCR7+CD45RO-CD28+), central memory (CM; CCR7+ CD45RO+ CD28+), transitional memory (TM; CCR7-CD45RO+CD28+), effector memory (EM; CCR7-CD45RO+CD28-), and effector (E; CCR7-CD45RO-CD28-; **Figure 4A**) cells. Among these subpopulations, the frequencies of CD74+ CM (mean: healthy 7.2%, mild 23.4%, severe 28.0%) and CD74+ TM (mean: healthy 8.8%, mild 17.8%, severe 25.0%) significantly increased upon SARS-CoV-2 infection, whereas frequencies of CD74+ effector cells decreased (mean: healthy 11.4%, mild 6.1%, severe 6.7%; **Figure 4B**). A proportion of the naïve and EM subpopulations also expressed CD74, yet no significant effect of the SARS-CoV-2 infection was observed. Changes in frequencies of CD74+ convCD4+ T cells expressing the co-receptors CXCR2 or CXCR4 upon SARS-CoV-2 infection are shown in **Figure 4C**. Frequencies of CXCR2+ cells were elevated on naïve, CM, and TM subpopulations in COVID-19 compared to healthy controls. The majority of CD74+ convCD4+ T cells at all stages of differentiation co-expressed the CXCR4 molecule, but increased frequencies of CXCR4+ CD74+ convCD4+ T cells in COVID-19 were only detected in cells with a naïve phenotype (mean: healthy 83.8%, mild: 82.1%, severe: 93.3%; **Figure 4C**, lower panel). Interestingly, when analyzing absolute numbers of CD4+ T cell subpopulation, we observed significantly increased numbers of TM and EM convCD4+ T cells expressing CD74 in the blood of COVID-19 patients (**Supplement 3A**), despite their lymphopenia. Thus, SARS-CoV-2 infection results in a significant upregulation of CD74 and its co-receptor CXCR2 on convCD4+ T cells with a memory phenotype.

### Expression of CD74 is enhanced on memory CD8+ T cells, but its co-receptors rather on effector CD8+ T cells in COVID-19

**Figure 5:**
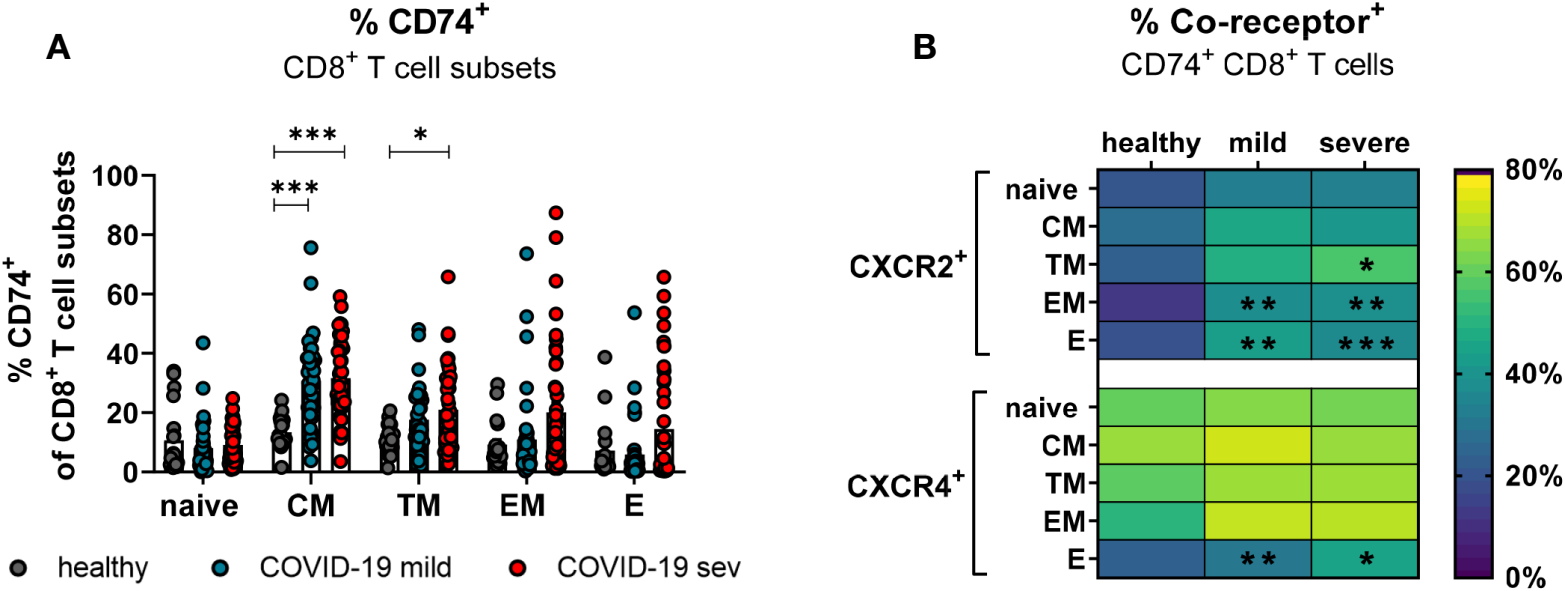
CD74 and co-receptor surface expression on CD8^+^ T cell subsets. Naïve (CCR7^+^CD45RO^−^CD28^+^), central memory (CM, CCR7^+^CD45RO^+^CD28^+^), transitional memory (TM, CCR7^−^CD45RO^+^CD28^+^), effector memory (EM, CCR7^−^CD45RO^+^CD28^−^), and effector (E, CCR7-CD45RO^−^CD28^−^) CD8^+^ T cell subpopulations were characterized by using CD45RO, CCR7, and CD28. Percentages of CD74^+^ CD8 T cell subsets **(A)** from mildly or severely ill COVID-19 patients and healthy donors were analyzed by flow cytometry. Frequencies of co-receptor positive CD74^+^ CD8^+^ T cell subsets are depicted in **(B)** for CXCR2^+^ and CXCR4^+^. Each dot represents an individual patient. Statistically significant differences are indicated by asterisks (* < 0.05; ** < 0.01; *** < 0.001).

CD8+ T cells with an effector phenotype can eliminate virus-infected cells and contribute significantly to the control of various viruses. In order to determine which subpopulations of CD8+ T cells might be responsive to MIF during COVID-19, the expression of CXCR2 and CXCR4 on CD74+ CD8+ T cells was analyzed. The differentiation of CD8+ T cell subpopulations was characterized by CD45RO, CCR7, and CD28. Even though SARS-CoV-2 infection led to slightly increased frequencies of all CD74+ CD8+ T cell subpopulations, only differences in CM (mean: healthy 13.4%, mild 30.8%, severe 31.6%) and TM (mean: healthy 11.0%, severe 20.9%) cells reached statistical significance compared to healthy controls (**Figure 5A**). CXCR2 was only expressed on a small proportion of CD8+ T cells at different stages of differentiation in healthy donors. SARS-CoV-2 infection led to a significant increase in the percentages of CXCR2+ CD74+ CD8+ T cells mainly in the effector (mean: healthy 20.5%, mild 44.1%, severe 37.9%) and EM (mean: healthy 13.5%, mild 38.3%, severe 39.5%) subpopulations (**Figure 5B**, upper panel). The frequency of CD74+CD8+ T cells expressing CXCR4 in healthy controls was highest on CM cells (mean: 67.8%) and lower for the other CD8+ T cell subpopulations (mean: naïve 61.1%, TM 59.9%, EM 51.7%, E 24.6%). Values of CD74+ CD8+ T cell frequencies expressing CXCR4+ were considerable variable in both groups of infected patients and only frequencies of CXCR4-expressing E CD74+ CD8+ T cells from COVID-19 patients showed significant increases compared to the healthy controls (healthy: 24.6%, mild: 31.8%, severe: 46.0%; **Figure 5B**, lower panel). Analysis of absolute numbers of CD8+ T cell subsets in the blood showed a reduction in CD74+ cell counts naive and TM phenotypes, but increased numbers of CD74+ EM and E cells in COVID-19 patients (**Supplement 3B**). These changes in numbers of CD74+ T cell subsets correlated with SARS-CoV-2-induced changes in the frequencies of CD8+ T cell subsets observed in our previous study ((23) **Figure 2C**).

Taken together, CD8+ T cells with effector phenotype enhanced their CD74 and co-receptor expression after SARS-CoV-2 infection.

### Kinetic analysis of CD74 and co-receptor expression on T cells during COVID-19

**Figure 6:**
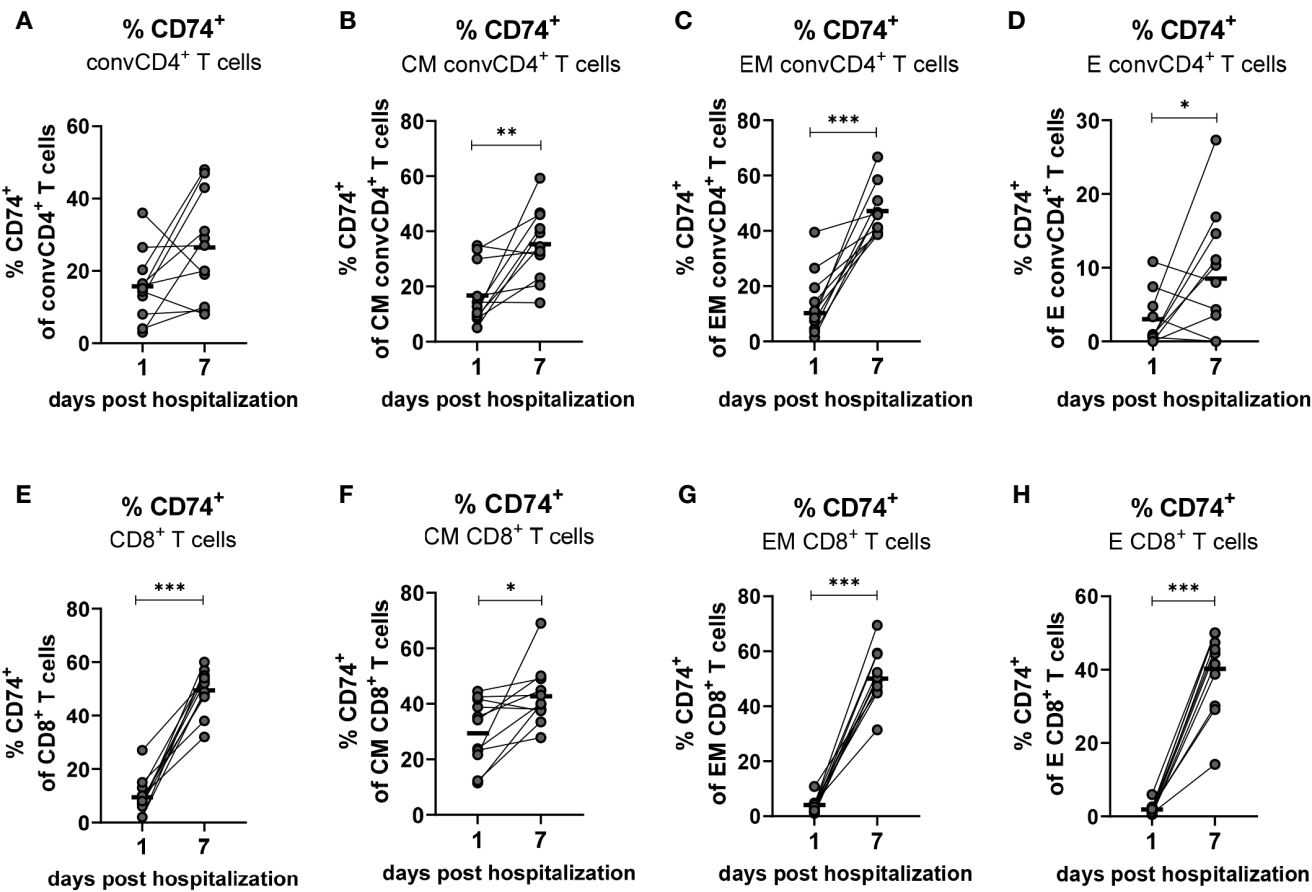
Kinetics of CD74^+^ on convCD4^+^ and CD8^+^ T cells. PBMCs from COVID-19 patients were isolated on the first and seventh day of hospitalization. central memory (CM, CCR7^+^CD45RO^+^CD28^+^), effector memory (EM, CCR7^−^CD45RO^+^CD28^−^), and effector (E, CCR7-CD45RO^−^CD28^−^) CD8^+^ T cell subpopulations were characterized by using CD45RO, CCR7, and CD28. Frequencies of CD74^+^ convCD4^+^ T cell subpopulations **(A–D)** as well as CD8^+^ T cell subpopulations **(E–H)** were determined by flow cytometric analysis. Each dot represents an individual patient and lines connect values of an identical patient on day 1 and 7 of hospitalization. Mean values of are indicated by bars. Statistically significant differences between time points are indicated by asterisks (* < 0.05; ** < 0.01; *** < 0.001).

Based on aforementioned changes in MIF receptor complex expression on different T cell subpopulations, our next aim was to analyze longitudinal changes of the MIF receptor expression on convCD4+ and CD8+ T cells on the day of COVID-19 hospital admission and on day 7 of hospitalization. Blood was drawn from hospitalized patients with mild and severe COVID-19 within the first 24 hours after admission to the hospital (day 1) as well as on the seventh day of hospitalization (day 7), and isolated T cells were analyzed for the expression of the MIF receptor CD74. Even though percentages of CD74^+^ convCD4^+^ T cells increased in most patients from day 1 to 7 the overall differences were not significant (mean: 15.7% on day 1, 26.5% on day 7; **Figure 6A**). When we analyzed subpopulations of convCD4^+^ T cells the results did not reach significance. However, frequencies of CM, EM, and E convCD4+ T cells expressing CD74 significantly increased from day 1 to 7 post hospital admission in COVID-19 (**Figures 6B–D**), highlighting the importance of a sub-population-specific assessment. Most prominent was the increase in CD74^+^ EM cells, which was found in virtually all analyzed patients (mean: 10.5% on day 1, 47.2% on day 7; **Figure 6C**).

While only 9.5% of the CD8+ T cells from COVID-19 patients were positive for the MIF receptor CD74 on the day of hospital admission, the population of CD74-expressing CD8^+^ T cells increased to a mean of 49.5% on day 7 (**Figure 6E**). This strong increase in CD74 expression on CD8+ T cells during COVID-19 was found in virtually all analyzed patients, and was also demonstrated for the cell populations EM (mean: 4.0% on day 1, 50.0% on day 7; **Figure 6G**) and E (mean: 1.9% on day 1, 40.2% on day 7; **Figure 6H**). It was less pronounced but still significant for CM CD8^+^ T cells (mean: 29.7% on day 1, 43.11% on day 7; **Figure 6F**). Taken together, we demonstrate that the disease progression of COVID-19 is associated with a substantial increase in the expression of CD74 on the CM, EM and E subpopulations of total convCD4+ and CD8+ T cells.

### Production of cytotoxic molecules in CD74+ T cells

**Figure 7:**
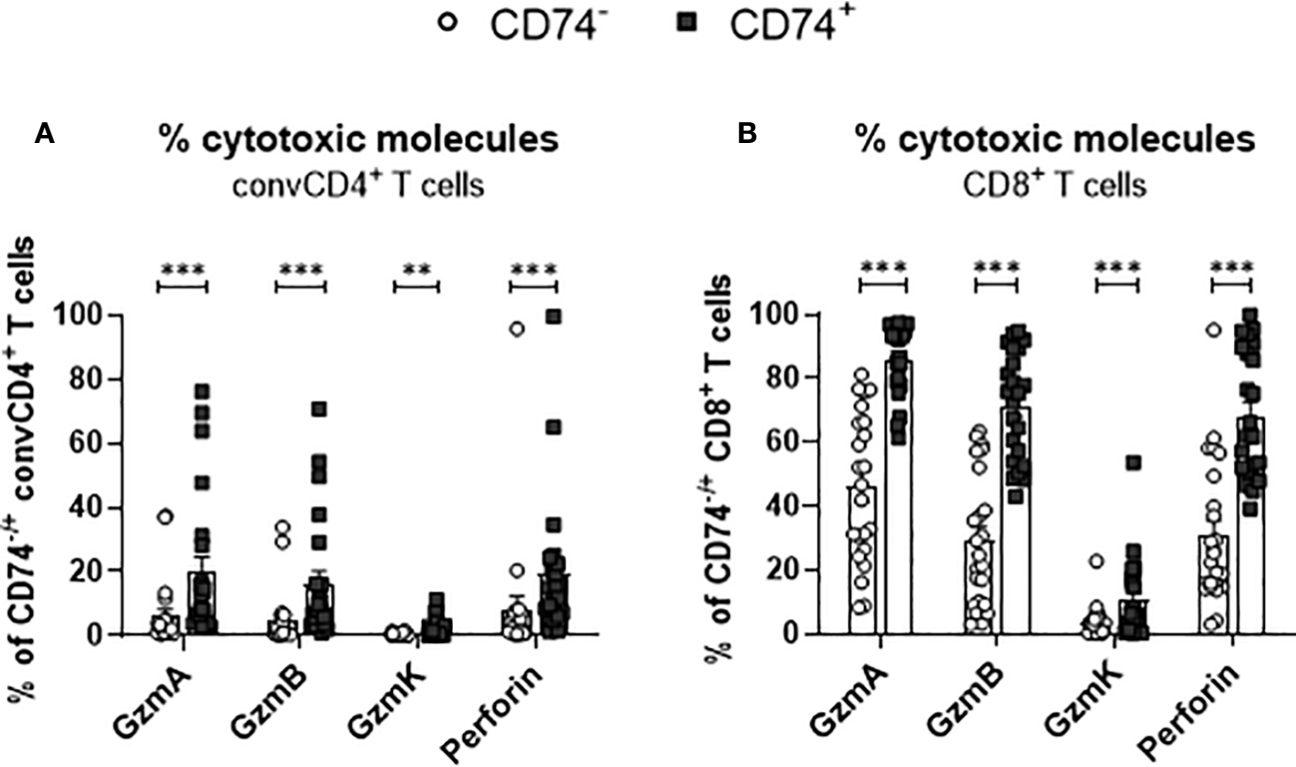
Production of cytotoxic molecules by CD74^−^ and CD74^+^ T cells. ConvCD4^+^ T cells **(A)**, and CD8^+^ T cells **(B)**, either CD74^−^ (white dots) or CD74^+^ (black squares), from COVID-19 patients were analyzed for the intracellular expression of the cytotoxic molecules GzmA, GzmB, GzmK, and Perforin. Each dot represents an individual patient. Statistically significant differences are indicated by asterisks (** <0.01; *** <0.001).

Differentiated T cells produce cytotoxic granules and mediate the elimination of target cells. Granzymes and perforin are the main cytotoxic effector molecules of T cells. In a previous study, we observed that SARS-Cov-2 infection results in the production of cytotoxic molecules in CD8+ T cells (23). To interrogate if an expression of the MIF receptor CD74 is associated with the expression of cytotoxic molecules in T cells, convCD4+ T cells as well as CD8+ T cells from COVID-19 patients were analyzed. ConvCD4+ T cells or CD8+ T cells were stratified into CD74- and CD74+ cells and intracellular levels of Granzyme A (GzmA), Granzyme B (GzmB), Granzyme K (GzmK), and Perforin were quantified. In accordance with previous reports (23), the overall frequencies of convCD4+ T cells producing cytotoxic molecules were low in COVID-19 patients. Intriguingly, percentages of CD74+ CD4+ T cells which expressed the cytotoxic molecules GzmA, GzmB, GzmK, and Perforin were significantly higher in comparison to CD74- convCD4+ T cells (mean CD74+: GzmA 19.8%, GzmB 16.0%, GzmK 1.9%, Perforin 19.4% and mean CD74-: GzmA 6.0%, GzmB 4.7%, GzmK 0.3%, Perforin 8.1%, **Figure 7A**). For CD8+ T cells, the overall frequencies of cells that produced cytotoxic molecules were much higher in comparison to convCD4+ T cells, independent of their surface expression of CD74 (**Figure 7B**). Nevertheless, the comparison of CD74- and CD74+ CD8+ T cells revealed significantly higher percentages of cells producing GzmA, GzmB, GzmK, and Perforin among the CD74+ CD8+ T cells (mean CD74+: GzmA 86.0%, GzmB 71.4%, GzmK 10.3%, perforin 68.3% and mean CD74-: GzmA 46.0%, GzmB 29.1%, GzmK 3.3%, perforin 30.8%, respectively; **Figure 7B**). Thus, in COVID-19 patients, CD74+ T cells more frequently produce cytotoxic molecules in comparison to their CD74- counterparts. For CD8+ T cells, the CD74 molecule in combination with differentiation markers may be suitable as a biomarker for activated and possible harmful effector T cells in COVID-19 pathology.

### COVID-19 leads to enhanced proliferation of CD74+ T cells

**Figure 8:**
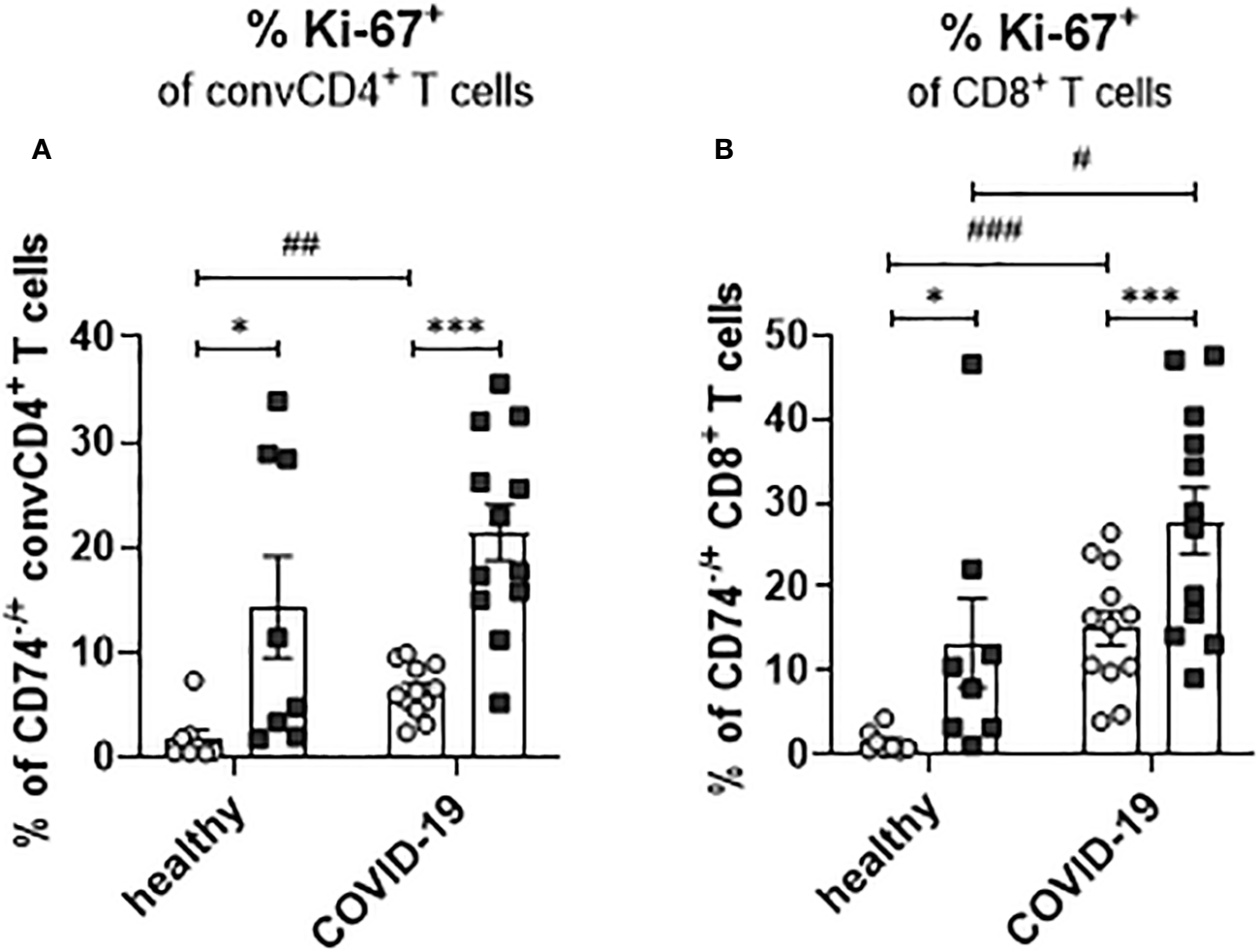
Proliferation of CD74^−^ and CD74^+^ T cells. ConvCD4^+^ T cells **(A)**, and CD8^+^ T cells **(B)**, either CD74^−^ (white dots) or CD74^+^ (black squares), from COVID-19 patients or healthy individuals were analyzed for the intracellular expression of proliferation marker Ki-67. Each dot represents an individual patient. Statistically significant differences are indicated by asterisks or hashs (< 0.05; **< 0.01; ***< 0.001, paired *t* test; ^#^ < 0.05; ^##^< 0.01; ^###^< 0.001).

During viral infection, bystander memory T cells are re-activated and increase their frequencies to level otherwise observed for effector T-cell populations. To assess whether a SARS-CoV-2 infection leads to changes in the proliferation of activated T cells and to test a possible association of enhanced proliferation with the expression of the MIF receptor CD74, frequencies of Ki-67+ convCD4+ T cells and CD8+ T cells were analyzed. ConvCD4+ T cells as well as CD8+ T cells from COVID-19 patients or healthy controls were stratified into CD74+ and CD74- cells and the percentage of T cells expressing intracellular Ki-67, a surrogate marker for proliferation (27), was detected. For both convCD4+ and CD8+ T cells, the percentages of Ki-67+ cells were always significantly higher in the population of the CD74+ cells compared to the CD74- ones (**Figures 8A, B**). This was independently found in healthy controls and COVID-19 patients, suggesting that MIF receptor expression might be associated with T-cell proliferation. In addition, SARS-CoV-2 infection increased percentages of proliferating CD4+ and CD8+ T cells in the CD74- subpopulations, as well as for CD8+ T cells in the CD74+ population (**Figures 8A, B**). Thus, the comparison of CD74+ T cells and their CD74- counterparts revealed that CD74+ CD4+ and CD8+ T cells proliferated more intensively than CD74- T cells, and that this proliferation was further enhanced upon SARS-CoV-2 infection. The expansion, proliferation, and production of cytotoxic molecules in differentiated T cell populations implies an association of MIF receptor expression with activation of bystander T cells.

## Discussion

The balance between the elimination of infected cells and maintaining the functions of the infected organ is a critical aspect of successful antiviral defense - especially when it comes to cytotoxic immune responses. In cases of excessive immune responses, the progressive expansion of virus-specific cytotoxic T lymphocytes leads to inflammation and damage of the affected organ - in the worst situation culminating in organ failure. Besides antigen-specific cell clones, viral infections induce the antigen-independent activation of bystander T cells. In COVID-19, the quantity of activated bystander T cells exceeds the quantity of SARS-CoV-2-specific T cells by orders of magnitude (21). Upon activation, bystander T cells acquire effector functions and can become potentially pathogenic cells (28). Expanded activated bystander CD8+ T cells were also observed during infections with HAV (29), HBV (30, 31), IAV (32) and HIV (33, 34). It was shown that activated bystander CD8+ T cells can produce pro-inflammatory cytokines and cytotoxic molecules such as perforin and GzmB (29, 35, 36). The expansion of bystander CD4+ T cells was also observed during the immune response against LCMV and HSV (22, 37). It was previously shown that a TCR-independent expansion of bystander CD4+ and CD8+ T cells and their effector functions can contribute to organ pathology and disease progression during autoimmune and inflammatory diseases (38, 39). The expansion of bystander CD8+ T cells is regulated by type I IFN, IL-12, IL-15, and IL-18, while the stimulation with IL-2, IL-12, IL-18 and IL-23 is necessary for the expansion of bystander CD4+ T cells (40). In our study, we observed the expression of CD74 on activated (CD44+) CD4+ and CD8+ T cells. Most of these cells were not specific for SARS-CoV-2 antigens. Simultaneously, enhanced concentrations of MIF in the serum of COVID-19 patients were observed. MIF is produced and stored in intracellular granules and can be released in response to different kinds of stress (41). In previous studies, an enhanced MIF concentration was associated with disease progression in SARS-CoV-2-infected patients (4). MIF is a multipotent cytokine which is associated with inflammatory and autoimmune diseases (42, 43). Moreover, in arthritis, myelitis, EAE, and mouse autoimmune models, MIF deficiency was associated with reduced pathologies and in some of these models a reduction in T cell activation was observed (44). Interestingly, different inflammatory lung diseases are associated with high concentrations of MIF (45–47). Additionally, it was shown that MIF also induces the activation and migration of monocytes and T cells in inflamed atherosclerotic arteries (12). The biological role of the soluble form of the CD74 molecule is not fully understood. We observed an enhanced concentration of sCD74 in the plasma of patients with severe COVID-19. It has been suggested that sCD74 abrogates MIF-mediated pro-inflammatory effects and leads to a reduction in immunopathology (48). However, in inflammatory lung disease, high concentrations of sCD74 were associated with pore prognosis, lung injury, and disease progression (49). Since we have contradictory results from a low number of studies, additional experiments are necessary to understand the biological role of MIF/sCD74 and membrane-bound CD74. Another immunoregulatory pathway that is mediated by MIF is the modulation of Tregs. In human blood, more than 20 percent of the Tregs express CD74 (**Figure 3B**). After SARS-CoV-2 infection, significantly more Tregs express CXCR2 and CXCR4 (**Figures 3D, E**). This can make Tregs more susceptible to MIF regulation. However, the effects of MIF on Tregs is poorly understood until now. To our knowledge, the effect of MIF on bystander T cells has also not been characterized so far. Bystander T cells are antigen-experienced cells that constitutively express CD44 (50, 51). CD44 is a cell surface glycoprotein, which is able to transduce the signal from CD74 upon recognition of MIF (13). In our study, we determined the possible susceptibility of bystander T cell populations to MIF by analyzing the expression of the receptor CD74 on the cell surface. During COVID-19, T cells show an enhanced expression of CD74. Additionally to the constitutively expressed CD44, a part of the CD74+ T cells also start to express the co-receptor molecules CXCR2 and CXCR4. All these data support our hypothesis that MIF is able to regulate bystander T cell activation. The binding of MIF to CD74 can provide signaling by the constitutively expressed CD44 or by the inducible co-receptor molecules CXCR2 or CXCR4 (12, 13). We observed that higher frequencies of T cells from patients with severe COVID-19 expressed CD74 (**Figure 2C**). This expression can make T lymphocytes more susceptible to MIF signaling and to CD74-dependent activation, since also the signaling coreceptor molecules CXCR2 and CXCR4 were enhanced on T cells in COVID-19 patients (**Figures 2E, F**). Analyzing absolute numbers of T cell subpopulations confirmed that COVID-19 patients have higher numbers of CD74+ effector T cells in the blood, which was especially true for effector CD8+ T cells (**Supplement 3B**). This at least correlated with disease severity, as it was more pronounced in the group of patients with severe COVID-19. Thus, it seems likely that the bystander T cell population develops a sensitivity to MIF during acute infection. Analyses of the T cell subpopulations in the early phase of SARS-CoV-2 infection showed that T cells with a central memory phenotype were the population with the highest expression of CD74. CM cells are long-lived cells with the ability to proliferate without specific antigen stimulation. Later during infection, T cell populations with EM and effector phenotypes also vigorously enhanced the expression of CD74. Moreover, subpopulations of CD74+ T cells harbored surrogate markers of proliferation and produced cytotoxic molecules. However, in the case of bystander cells during COVID-19, these effector populations were not specific for SARS-CoV-2 peptides, so the role of these cells in the control of virus is still elusive. The activation, expansion, and accumulation of bystander CD8+ T cells in infected organs may be a key factor of immunopathology in the lungs of COVID-19 patients. The pathogenetic role of bystander T cells was previously observed in some autoimmune diseases (52, 53). MIF signaling through its receptor complex including CD74 and other co-receptor molecules may have an effect on the large population of bystander T cells. Experimental and clinical studies on treatments targeting MIF and its receptors demonstrate efficacy in the therapy of rheumatoid arthritis (RA) and systemic lupus erythematosus (SLE). MIF and its receptor CD74 are interesting therapeutic targets for the systemic regulation of activated T cells. A broad spectrum of low molecular drugs, peptides, and antibodies directed against MIF or CD74 may be tested for the modulation of CD74-expressing T cells during inflammatory immunopathologies in different organs (54). Inhibitory treatments directed at bystander T cells and the MIF system are possible future immunotherapies, which may be effective for the reduction of disease severity and the protection of infected organs during viral infections.

## Data availability statement

The original contributions presented in the study are included in the article/**Supplementary Material**. Further inquiries can be directed to the corresponding authors.

## Ethics statement

The studies involving humans were approved by Ethics Committee of the Medical Faculty of the University Hospital Essen (ethics vote 20-9216-BO). The studies were conducted in accordance with the local legislation and institutional requirements. The participants provided their written informed consent to participate in this study.

## Author contributions

JW, MT, ML, DY, NB, TiW, UD, and GZ contributed to conception and design of the study. JW, AB, KP, ZK, TaW, DM, and GZ contributed to investigations, methodology, and data analysis. JW, ZK, MT, OW, ML, DM, IK, FH, UD, and GZ wrote and edited the manuscript. All authors contributed to the article and approved the submitted version.
