# Supplementary material for: Macrophage migration inhibitory factor receptor CD74 expression is associated with expansion and differentiation of effector T cells in COVID-19 patients
Source: Front Immunol. 2023 Oct 25;14:1236374. doi: 10.3389/fimmu.2023.1236374 (PMC10631787; doi:10.3389/fimmu.2023.1236374)
Supplement: Supplementary file 1 [file DataSheet_1.docx]

|  | Disease course | |  |
| --- | --- | --- | --- |
|  | mild | severe |  |
| Number of Patients | 39(18F, 21M) | 42(13F, 29M) |  |
| Age(median) | 64 | 54 |  |
|  |  |  |  |
| Median laboratory findings and range |  |  |  |
|  |  |  |  |
| White cell count-per µl | 5690(1810-17500) | 8210(2410-46490) |  |
| Red cell count-per pl | 4.14(3.1-5.59) | 4.04(2.48-5.18) |  |
| Lymphocyte count-per µl | 1030(410-3290) | 880(310-29680) |  |
| Neutrophile count-per µl | 3810(1260-1296) | 6850(950-16650) |  |
| Platelet count-per nl | 196(78-409) | 224(46-512) |  |
| Monocytes count-per µl | 400(100-1150) | 470(130-1110) |  |
| Hemoglobin-g/dl | 12.4(8.9-15.7) | 115.5(7.4-14.9) |  |
| Haematocrit l/l | 0.358(0.33-0.44) | 0.337(0.222-0.416) |  |
| C-reactive protein - mg/dl | 5.7(0.8-23.4) | 11.2(0.5-32.8) |  |
| Lactat dehydrogenase - U/L | 366(225-644) | 428(130-998) |  |
| Aspartate aminotransferase - U/L | 50(19-219) | 48(10-644) |  |
| Alanine aminotranserase - U/L | 36(10-204) | 35(12-483) |  |
| Gamma-glutamyl transferase  - U/L | 31.5(15-286) | 58(7-419) |  |
| Total Bilirubin - mg/dl | 0.5(0.2-1.3) | 0.6(0.3-14.2) |  |
|  |  |  |  |
|  |  |  |  |
