# Supplementary material for: Macrophage migration inhibitory factor receptor CD74 expression is associated with expansion and differentiation of effector T cells in COVID-19 patients
Source: Front Immunol. 2023 Oct 25;14:1236374. doi: 10.3389/fimmu.2023.1236374 (PMC10631787; doi:10.3389/fimmu.2023.1236374)
Supplement: Supplementary file 2 [file DataSheet_2.pdf]

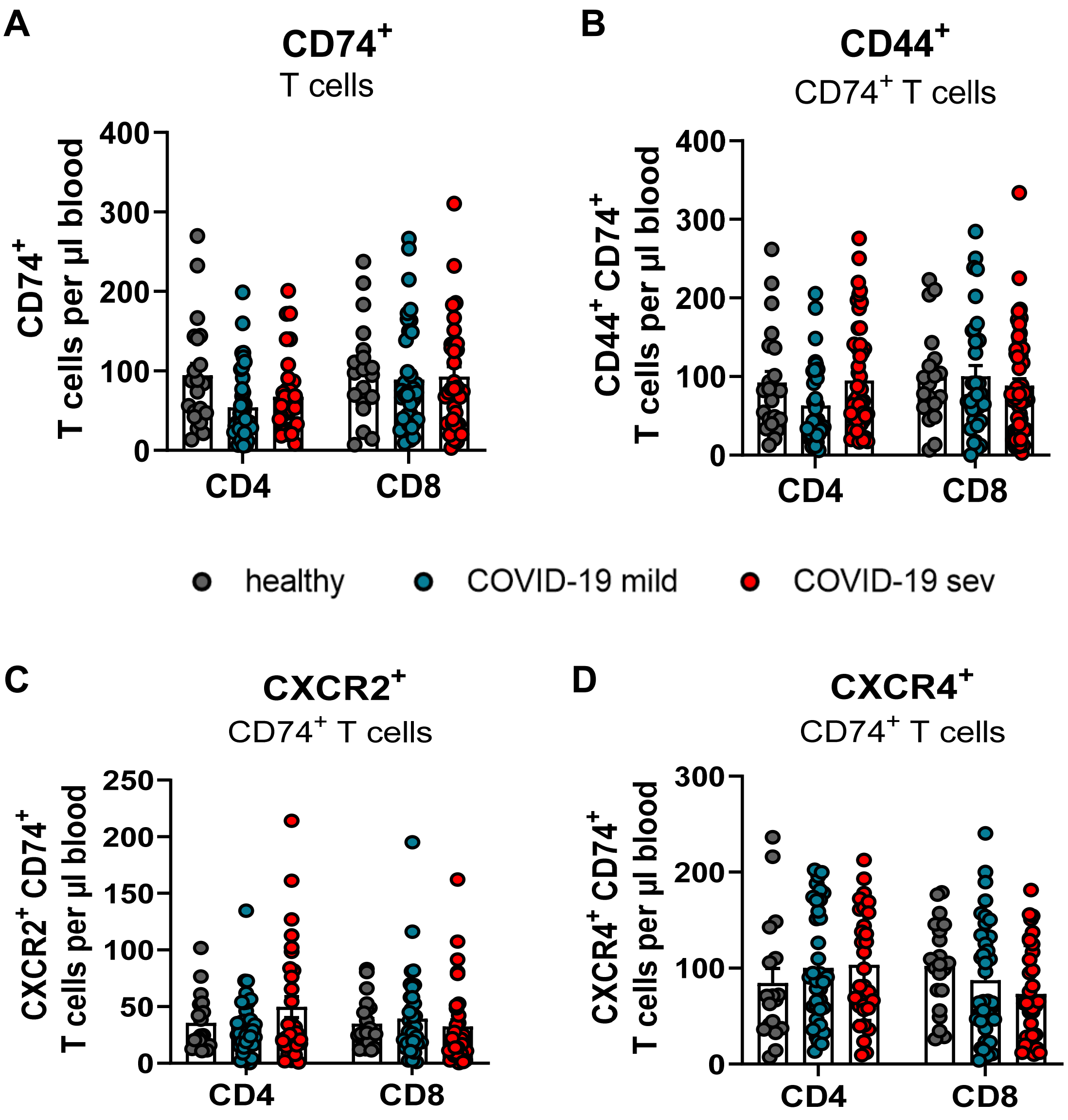

Supplement. 2. Concentrations of MIF receptor- and co-receptor positive T cells. Counts of $CD4^+$ and $CD8^+$ T cells per µl in the blood from mildly (mild) or severely (sev) diseased patients and healthy donors expressing CD74 (**A**), CD74 and CD44 (**B**), CD74 and CXCR2 (**C**) or CD74 and CXCR4 (**D**) were analyzed by flow cytometry. Each dot represents an individual patient.
