# Supplementary material for: Macrophage migration inhibitory factor receptor CD74 expression is associated with expansion and differentiation of effector T cells in COVID-19 patients
Source: Front Immunol. 2023 Oct 25;14:1236374. doi: 10.3389/fimmu.2023.1236374 (PMC10631787; doi:10.3389/fimmu.2023.1236374)
Supplement: Supplementary file 3 [file DataSheet_3.pdf]

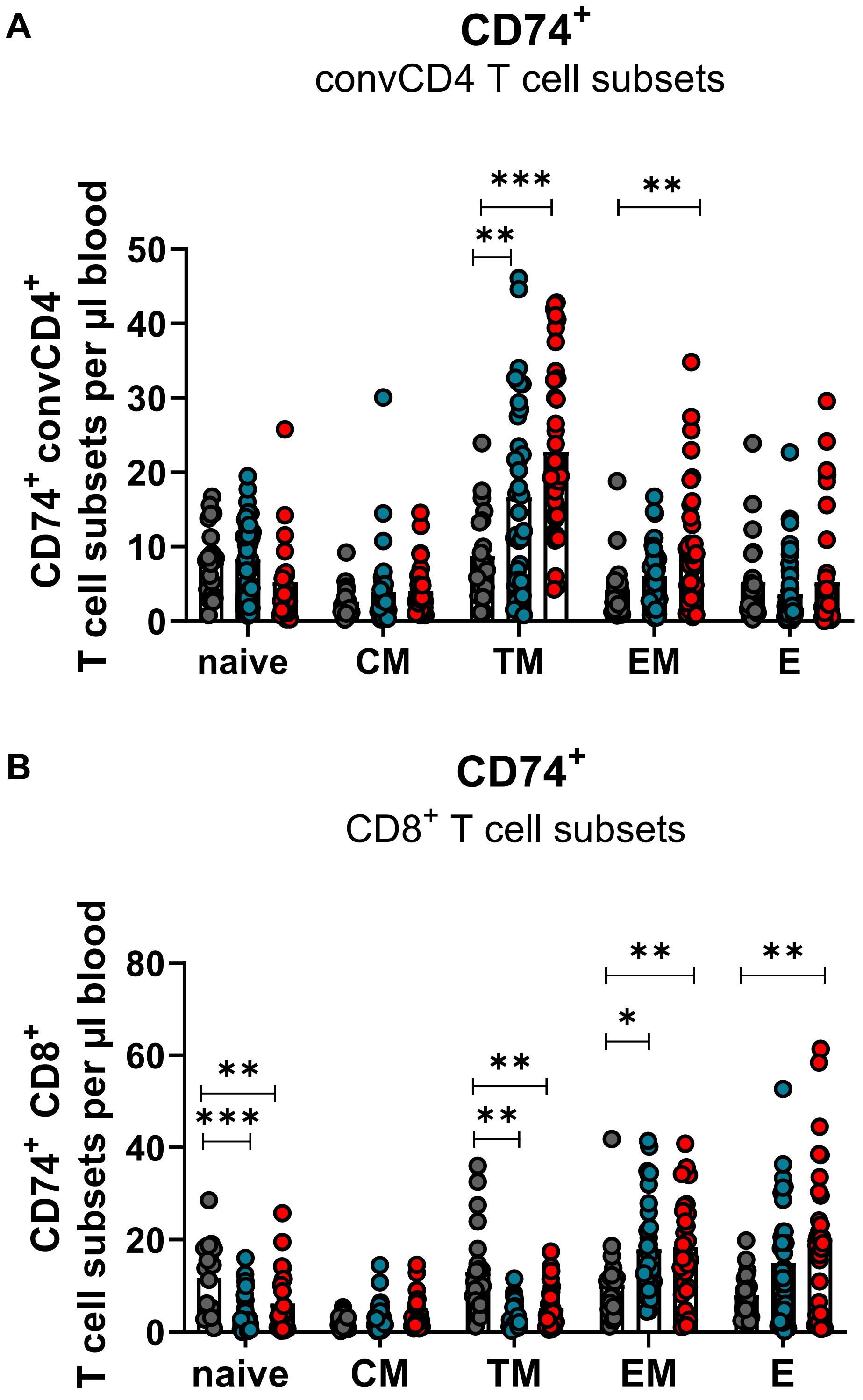

Supplement 3. Concentrations of CD74 expressed convCD4$^+$ and CD8+ T cells at different stages of differentiation.

Naïve (CCR7$^+$CD45RO$^-$CD28$^+$), central memory (CM, CCR7$^+$CD45RO$^+$CD28$^+$), transtional memory (TM, CCR7$^-$CD45RO$^+$CD28$^+$), effector memory (EM, CCR7$^-$CD45RO$^+$CD28$^-$), and effector (E, CCR7-CD45RO$^-$CD28$^-$) T cell subpopulations were characterized by using CD45RO, CCR7, and CD28. CD74$^+$ convCD4$^+$ (**A**) and CD8+ (**B**) T cell in different subsets were measured by flow cytometry and calculated. Each dot represents an individual patient. Statistically significant differences are indicated by asterisks (* < 0.05; ** < 0.01; *** < 0.001).
